# Evidence That Selenium Binding Protein 1 Is a Tumor Suppressor in Prostate Cancer

**DOI:** 10.1371/journal.pone.0127295

**Published:** 2015-05-18

**Authors:** Emmanuel Ansong, Qi Ying, Dede N. Ekoue, Ryan Deaton, Andrew R. Hall, Andre Kajdacsy-Balla, Wancai Yang, Peter H. Gann, Alan M. Diamond

**Affiliations:** 1 Department of Pathology, University of Illinois at Chicago, Chicago, Illinois, United States of America; 2 University of Illinois at Chicago, Chicago, Illinois, United States of America; 3 College of Food Science and Technology, Nanjing Agricultural University, Nanjing, China; 4 Department of Pathology, Xinxiang Medical University, Xinxiang, China; Rush University Medical Center, UNITED STATES

## Abstract

Selenium-Binding Protein 1 (SBP1, SELENBP1, hSP56) is a selenium-associated protein shown to be at lower levels in tumors, and its lower levels are frequently predictive of a poor clinical outcome. Distinguishing indolent from aggressive prostate cancer is a major challenge in disease management. Associations between SBP1 levels, tumor grade, and disease recurrence following prostatectomy were investigated by duplex immunofluorescence imaging using a tissue microarray containing tissue from 202 prostate cancer patients who experienced biochemical (PSA) recurrence after prostatectomy and 202 matched control patients whose cancer did not recur. Samples were matched by age, ethnicity, pathological stage and Gleason grade, and images were quantified using the Vectra multispectral imaging system. Fluorescent labels were targeted for SBP1 and cytokeratins 8/18 to restrict scoring to tumor cells, and cell-by-cell quantification of SBP1 in the nucleus and cytoplasm was performed. Nuclear SBP1 levels and the nuclear to cytoplasm ratio were inversely associated with tumor grade using linear regression analysis. Following classification of samples into quartiles based on the SBP1 levels among controls, tumors in the lowest quartile were more than twice as likely to recur compared to those in any other quartile. Inducible ectopic SBP1 expression reduced the ability of HCT-116 human tumor cells to grow in soft agar, a measure of transformation, without affecting proliferation. Cells expressing SBP1 also demonstrated a robust induction in the phosphorylation of the p53 tumor suppressor at serine 15. These data indicate that loss of SBP1 may play an independent contributing role in prostate cancer progression and its levels might be useful in distinguishing indolent from aggressive disease.

## Introduction

Prostate cancer is the most prevalent type of cancer among men with estimates of over 240,000 new cancer cases and 33,000 deaths in the United States alone in 2011 [1]. The disease is primarily found among older men with approximately 91% of those diagnosed having tumors confined to the primary site or local lymph nodes [2]. Early detection of prostate cancer has improved dramatically but it is becoming increasingly clear that treatment is given to a large segment of patients whose disease was indolent, therefore having little impact on their morbidity or mortality [3]. Given the negative side effects associated with prostate cancer treatments such as radical prostatectomy, hormonal therapy, brachytherapy and other forms of radiation therapy, a major challenge in managing prostate cancer is finding reliable means to distinguish clinically significant disease from that which is indolent and better off not being treated. To contribute to this effort, we have focused on the Selenium Binding Protein 1 (SBP1, SELENBP1, hSP56), a selenium-containing protein that is expressed in a variety of tissue types, including the brain, prostate, lung, and intestine. The form of selenium in SBP1 is unknown as is the nature of its association: the selenium remains bound to the protein when electrophoresed in SDS acrylamide gels but dissociates at extremes of pH [4]. The function of SBP1 also has not been established, although it may be involved in intra-golgi transport [5], has been shown to regulate HIF-1α [6] and is associated with two different isoforms of von Hippel-Lindau protein interacting deubiquitinating enzyme 1, which indicates SBP1 may have functions in protein degradation [6,7].

Low SBP1 levels are associated with poor clinical outcome in several cancer types. This was first shown for lung adenocarcinomas, where low levels of SBP1 were strongly associated with poor survival [8]. Subsequently, low levels of SBP1 were similarly shown to be associated with the poor prognosis of ovarian [9], colon [10,11] and most recently hepatocellular carcinoma [12]. Little is known about SBP1’s role in prostate cancer, although it is highly expressed in normal human prostatic tissue [13]. Here, we report on the assessment of whether levels of SBP1 are indicative of the likelihood of biochemical recurrence of prostate cancer using outcome tissue microarrays, as well as studies to begin understanding SBP1’s biological role in cancer.

## Materials and Methods

The UIC Office for the Protection of Research Subjects has determined that this work does not meet the definition of human subject research. All data obtained from human tissue was analyzed anonymously.

### Immunohistochemistry

Prostate cancer outcome tissue microarrays were obtained from the Cooperative Prostate Cancer Tissue Resource (CPCTR), a multi-institutional consortium to bank prostatectomy tissue, as well as relevant patient data including demographics, surgical pathology and follow-up history, under a set of uniform guidelines. The CPCTR has collected more than 6,000 prostate cancer and control samples, the largest collection of this type of tissue available in this country with clinical and pathological outcomes [14,15]. The arrays used in this study include tissue cores of 0.6 mm diameter, in quadruplicate, from 202 men ("cases") who experienced biochemical recurrence (a single post-surgery PSA value above 0.4 ng/ml or two consecutive PSAs above 0.2 ng/ml) after prostatectomy and 202 non-recurrent controls matched on age at surgery (+/- 5 years), year of surgery, race, Gleason score (both primary and secondary) and pathological stage. Tissues on the slides were blocked with H_2_O_2_ Blocking Reagent (Abcam) for 30 minutes, treated with a protein blocking solution for 15 minutes at room temperature, rinsed and incubated with monoclonal mouse anti-SBP-1 antibody (Cat. # M061-3, MBL International) and the CK8/18 antibody (American Research Products), both at a titer of 1:100 for 60 minutes at room temperature. Slides were rinsed in TBST for 5 minutes, and then treated with anti-mouse Alexa Fluor 647 and anti-rabbit Alexa Fluor 488 polymer for 20 minutes at room temperature. Slides were rinsed in distilled water; nuclei were counterstained with DAPI, dehydrated through an alcohol gradient and mounted with Micromount (Leica Microsystems). SBP1 and CK8/18 were detected in stained cores by multispectral scanning using the Vectra quantitative imaging system (Perkin Elmer, Hopkinton, MA). Autofluorescence was removed from the images and epithelial (tumor) areas of each core were identified by CK8/18 staining using inForm (Perkin Elmer) machine learning algorithms. Manual editing removed benign epithelium from analysis. DAPI staining was recognized by the software as the nucleus of each cell, and the cytoplasmic signal was obtained by sampling the peri-nuclear area. Within the tumor areas, we exported both nuclear and cytoplasmic data from the SBP1 channel on a per-cell basis.

### Statistical analysis

The fluorescent intensities of nuclear and cytoplasmic SBP1 were assessed for normality and log-transformed for statistical analysis. Patients were grouped into three categories based on their Gleason grade (Category 1 = Gleason ≤6, Category 2 = Gleason 7(3+4) Category 3 = Gleason 7(4+3) or ≥8). Cores with Gleason scores 4+3 were assigned to Category 3, the highest Gleason group, because the mortality rates of patients with prostate cancer of Gleason score 4+3 is three-fold higher than 3+4 cancers Stark 2009 [16]. The association between SBP1 levels and Gleason category was assessed using the Wilcox Rank Sum test. Patient tissues were also assigned to quartiles based on SBP1 intensity among the control subjects. Conditional logistic regression models were fitted to estimate odds ratios and 95% confidence intervals for the risk of biochemical recurrence for each quartile of SBP1. The conditional models incorporated adjustment for case-control matching variables; additional models were fit with pre-surgical PSA as a covariate, since PSA was not a matching factor. All data obtained using human tissue was analyzed anonymously.

### Plasmid Construction

A doxycycline-inducible SBP1 expression construct was generated by the insertion of the *SBP1* open reading frame generated by PCR amplification using a previously generated SBP1 construct as the template [17] and ligation into the pRetroX-Tight-Pur vector (Clontech, Mountain View, CA). For amplification, a forward primer (5’- ATAGCGGCCGCTACAGCATGGCTACGA AAT-3’) and reverse primer (5’-ACGAATTCGCTCAAATCCAGATGTCAGAGC-3’) were used, the amplification product was digested with *NotI* and *EcoRI* and directionally ligated into the vector. The inducible expression system includes the pRetroX-Tight-Pur-TetOn-Advanced plasmid containing the Tet-On transactivator gene. The Tet-On gene codes for a protein that binds to the promoter region of the pRetroX-Tight-Pur plasmid and induces transcription of the gene downstream of the promoter in response to doxycycline. The SBP1 gene was inserted downstream of the doxycycline responsive promoter region in the pRetroX-Tight-Pur plasmid yielding the pRetroX-Tight-Pur-SBP1.

### Cell Culture

The HCT116 human colon carcinoma cell line was verified (Genetica DNA Lavoratories, Burlington, NC) and maintained in McCoy’s 5a medium (Mediatech, Manassas, VA), and the GP2-293 retroviral packaging line was maintained in DMEM medium (Life Technologies, Carlsbad, CA). All media was supplemented with 10% FBS, 100 U/mL penicillin, and 100ug/mL streptomycin and incubated at 37°C with 5% CO_2_. The selenium concentration of the serum used was determined to be 152 nM by graphite furnace atomic absorption spectrometry conducted at the Texas A&M Veterinary Diagnostic Laboratory at College Station, Texas. Both the Tet-On transactivator and the SBP1 expression constructs were packaged into retroviral particles by cotransfection of each plasmid with the p-VSV-G retroviral envelope expression construct into the GP2-293 packaging cell line, and used to infect HCT116 cells. Infected cells were clonally selected in 400ug/mL G418 (Sigma) and 1ug/mL puromycin (Sigma), expanded, and screened for inducible SBP1 expression by western blotting following treatment with 1.0ug/mL doxycycline for 48 hours.

### Western Blotting

Treated cells were harvested by lysis in 1x Cell Lysis Buffer (Cell Signaling, Danvers, MA) containing 1mM PMSF (Cell Signaling). Lysates were boiled in NuPAGE LDS Sample Buffer (Life Technologies) and 10x Reducing Agent (Life Technologies) for 5 minutes and applied to a 4–12% gradient Bis-Tris denaturing polyacrylamide gel (Life Technologies). After electrophoresis, proteins were transferred to an Immobilon-FL membrane (Millipore) via electroblotting. Antibodies against the following proteins were used: SBP1 and GPx-1(mouse, MBL International, Woburn, MA), p-P53, (rabbit, Cell Signaling), P53 (rabbit, Santa Cruz, Santa Cruz, CA), and β-actin (rabbit, Abcam, Cambridge, MA).

### Cell Proliferation and growth in semi-solid media

Proliferation was assessed using the FluoReporter Blue Fluorometric dsDNA Quantification Kit (Invitrogen) that is based on the use of a Hoescht 33258 fluorescent stain to measure the quantity of double stranded DNA present in each well of a 96 well plate as an indicator of the amount of live cells present at a given time point. 5x10^2^ cells were plated in each well of a black, clear bottom 96 well plate and treated as indicated (48, 96 and 144 hours). Culture medium was removed at the selected time points and the plates were immediately stored at -80°C until all time points were completed. Plates were thawed at room temperature and cells lysed using distilled water to extract cellular DNA. Hoechst 33258 dye was added to each well to stain DNA, and fluorescence was quantified using excitation and emission filters at 360nm and 460nm respectively. In order to determine the ability of SBP1 to influence growth in semi-solid media, cells induced to express SBP1 as well as SBP1-null cells were treated with doxycycline, mixed with media containing 0.4% agarose, and each group was plated in triplicate on 6 well plates containing 0.6% agarose in media. Each well contained 5x10^4^ cells, and colonies larger than 0.5mm were enumerated on day 15.

## Results

### Fluorescent imaging of SBP1 in human prostate cancer indicates strong nuclear localization and sporadic intraglandular expression

**Fig 1:**
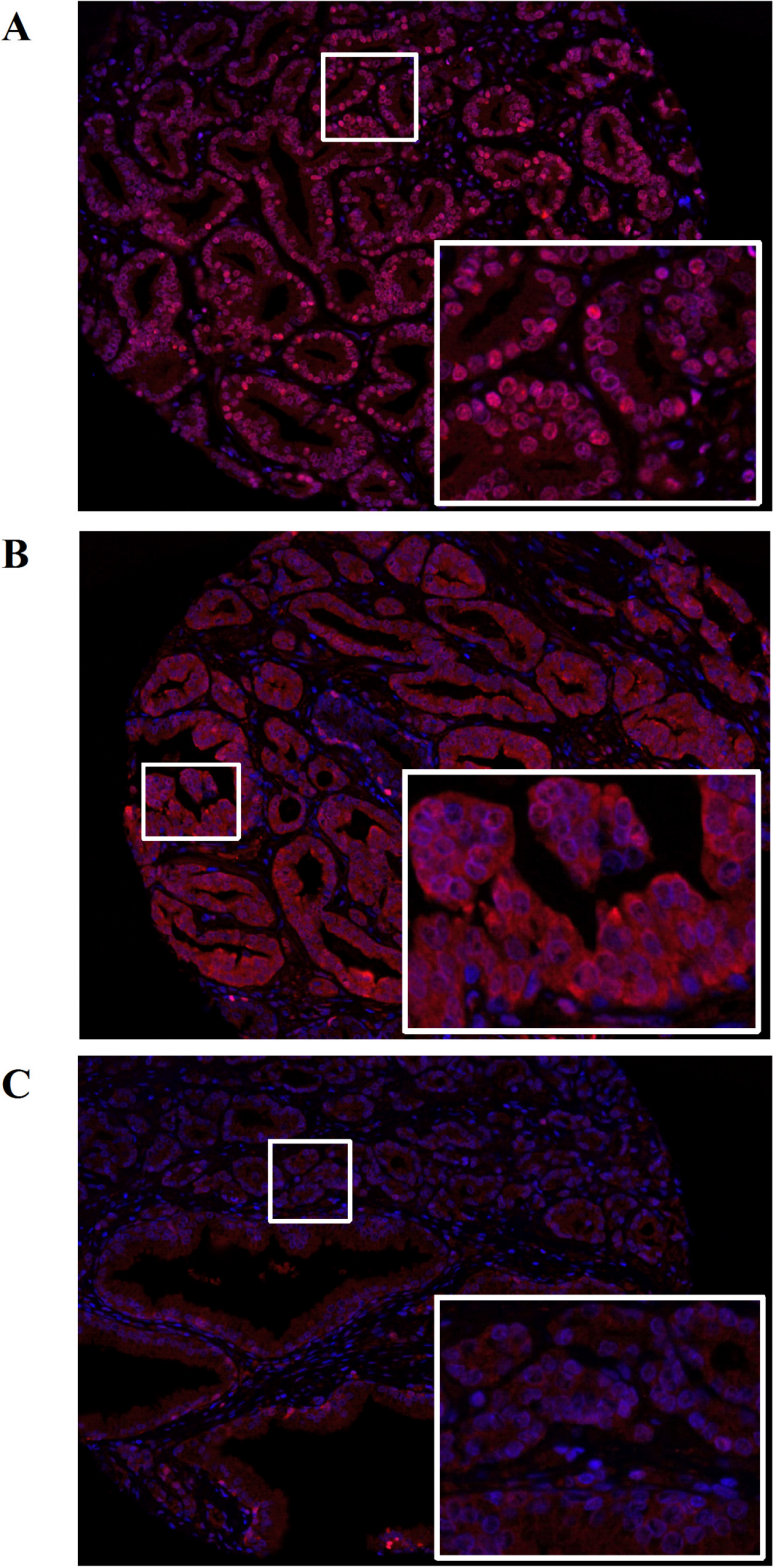
SBP1 levels differ in human prostate tissue. An example of the image of a core obtained from the outcome CPCTR tissue microarray stained with anti-SBP1 (red) antibodies followed by Alexa488 goat anti-rabbit and Alexa647 goat anti-mouse respectively. Nuclei were counterstained with DAPI (blue). A is an example of nuclear localized SBP1, B shows cytoplasmic SBP1, and C is an example of very low SBP1 expression in prostate tissue.

After poor quality and benign-only cores were excluded, the cohort for analysis included tissue from 168 case-control pairs. Examples of scanned cores from the microarray are presented in Fig 1. Highly variable nuclear SBP1 levels were often observed among epithelial cells in the same gland, with adjacent cells often showing varying staining intensity among the cells that were SBP1-positive (Fig 1A). In addition, prostate tissue with also varied with respect to cytoplasmic and nuclear staining, as exemplified in Fig 1B & 1C.

### The SBP1 nuclear to cytoplasmic ratio is inversely correlated with prostate cancer Gleason score

**Fig 2:**
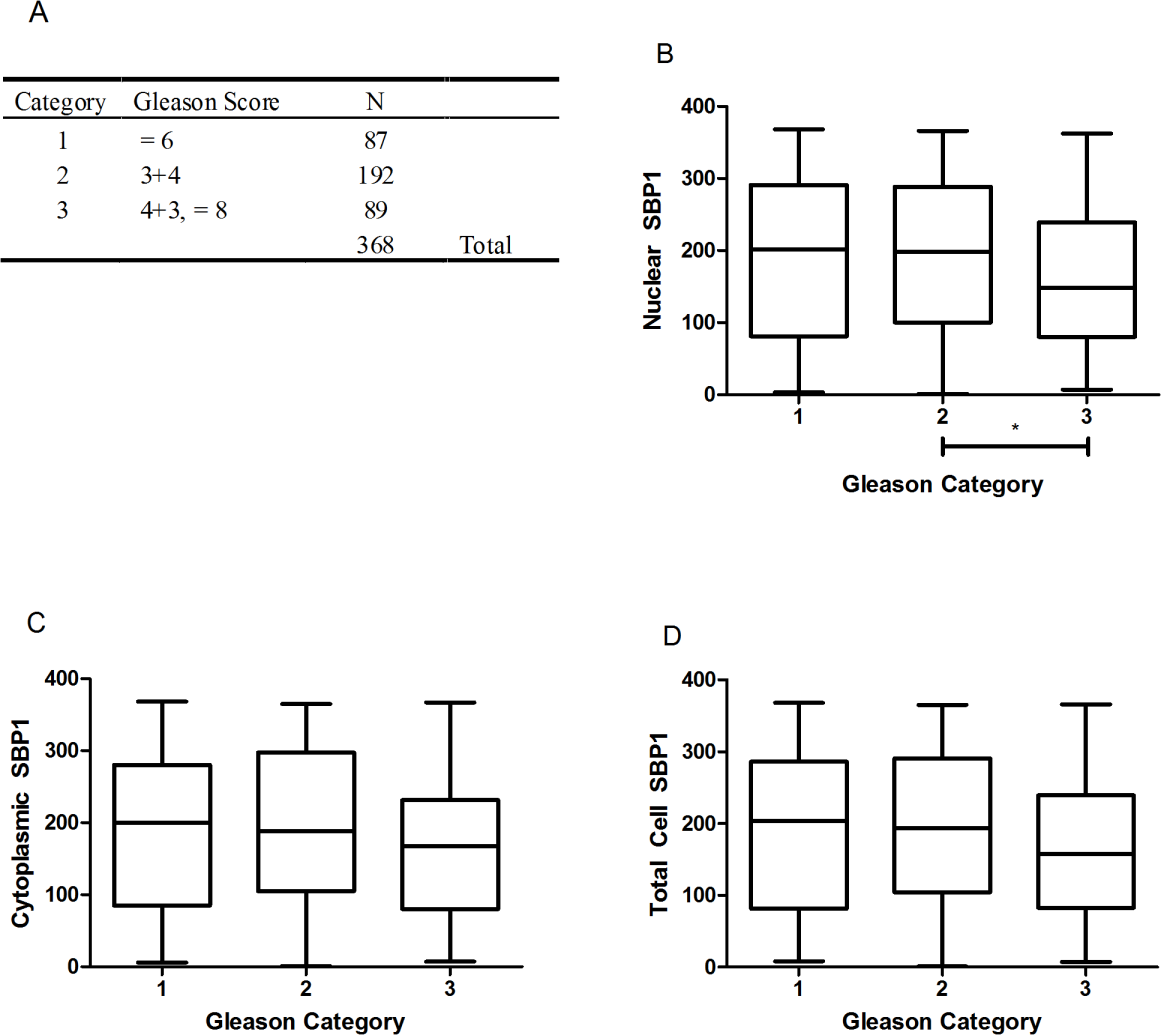
Lower SBP1 levels in the nucleus are significantly associated with advanced prostate cancer. Patients in the prostate cancer outcome tissue microarray were distributed into three categories (Gleason Category) based on Gleason score (A). Mean quantified tissue SBP1 was ranked for all patients and assigned a score from 1–368 based on relative SBP1 levels where the patient with the lowest tissue SBP1 received a score of 1, and the patient with the highest SBP1 levels received a score of 368. Box-and-whisker plots indicate the range of scores from patients in each category (vertical lines) and the range of the first and third quartile in each category (boxes). The horizontal line inside the box indicates the median. The association between SBP1 and Gleason Category was determined using SBP1 in the nucleus (B), cytoplasm (C) and total cell (D).

**Fig 3:**
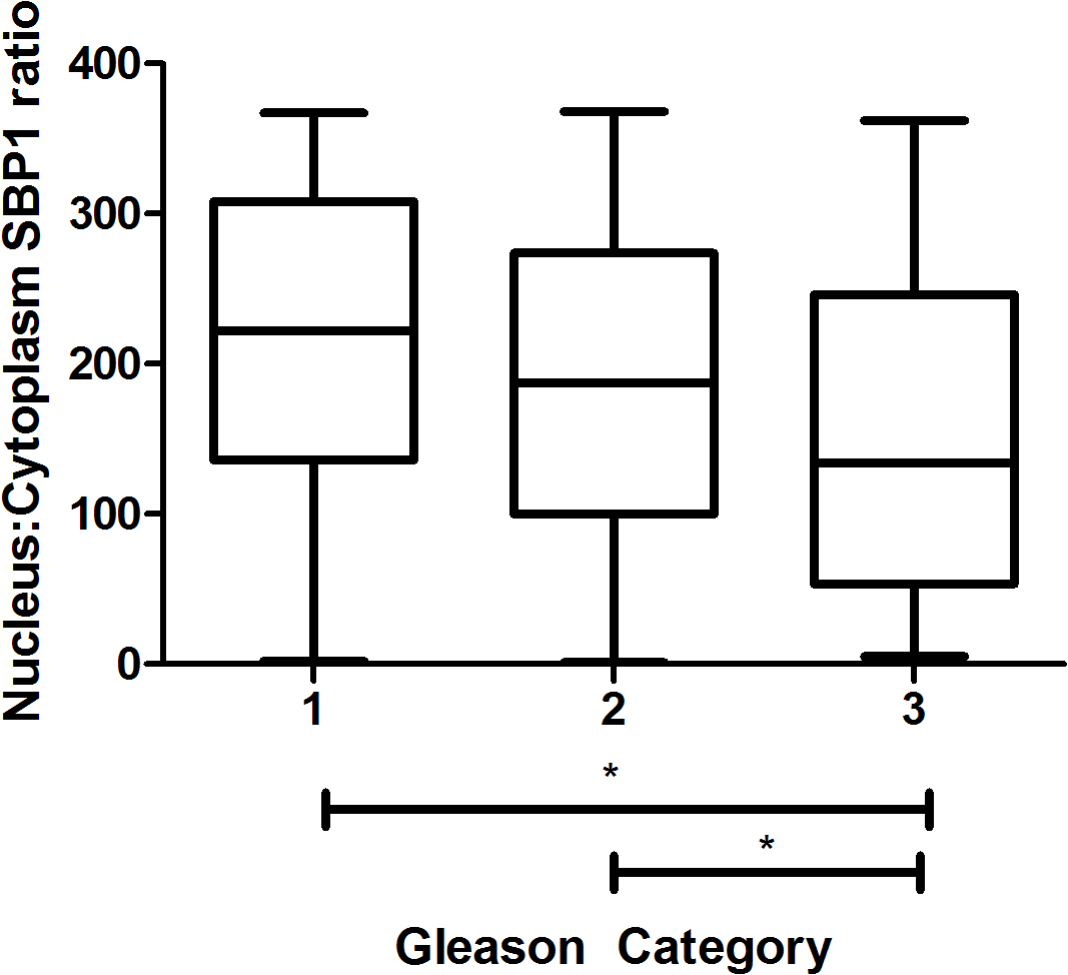
Lower nucleus:cytoplasm SBP1 ratio is significantly associated with advanced prostate cancer. Patients in the prostate cancer outcome microarray were distributed into three categories (Gleason Category) based on Gleason score (A). Mean quantified tissue SBP1 was ranked for all patients and assigned a score from 1–368 based on relative SBP1 levels where the patient with the lowest tissue nuclear:cytoplasmic ratio of SBP1 received a score of 1, and the patient with the highest ratio received a score of 368. Box-and-whisker plots indicate the range of scores from patients in each category (vertical lines) and the range of the first and third quartile in each category (boxes). The horizontal line inside the box indicates the median.

In order to assess the association between SBP1 levels and Gleason score, patients were grouped into three categories based on their Gleason grade (Category 1 = Gleason ≤6, Category 2 = Gleason 7(3+4) Category 3 = Gleason 7(4+3) or ≥8). Analysis of SBP1 levels in prostatic tissues indicated that the average nuclear SBP1 signal in each cell was significantly lower in Category 3 than in Category 2 (Fig 2). Additionally, the nuclear:cytoplasmic ratio of SBP1 was significantly lower in Category 3 vs. Category 1 (Fig 3). Cytoplasmic and total cell SBP1 levels were not significantly associated with Gleason score, nor was there a significant difference between Categories 1 and 3.

### Prostate cancer patients in the lowest quartile of SBP1 expression are significantly more likely to recur after radical prostatectomy

To determine if there was an association between SBP1 and biochemical recurrence, SBP1 levels in the nucleus, cytoplasm, and total cell were first analyzed using a paired t-test. No significant difference between recurrent cases and non-recurred controls was observed. To examine the possibility of a non-linear association between SBP1 expression and recurrence, the samples were alternatively distributed into quartiles based on tissue SBP1 levels among controls. Using this approach, logistic regression analysis of the interquartile odds of prostate cancer recurrence indicated that patients in the lowest quartile of nuclear SBP1 levels were significantly more likely to recur than those in any other quartile (Table 1). All estimates were adjusted for PSA, Gleason grade, stage, race, year of surgery and age at diagnosis.

**Table 1:** Patients whose tissue was in the lowest quartile of nuclear SBP1 are significantly more likely to recur after radical prostatectomy than patients whose tissues had higher nuclear SBP1.

	SBP1 levels by quartile			
	1 (Low)	2	3	4 (high)
	OR (95% CI)	OR (95% CI)	OR (95% CI)	OR (95% CI)
Nuclear SBP1	**1.00**	**0.36** (0.16, 0.81)	**0.44** (0.20, 0.99)	**0.34** (0.13, 0.91)
Cytoplasmic SBP1	**1.00**	**0.35** (0.16, 0.74)	**0.54** (0.24, 1.19)	**0.48** (0.19, 1.22)
Total SBP1	**1.00**	**0.24** (0.10, 0.56)	**0.59** (0.27, 1.26)	**0.28** (0.10, 0.77)
	**Quartile 2–4 vs. 1**			
	**OR** (95% CI)			
Nuclear SBP1	**0.43** (0.22–0.84)			
Cytoplasmic SBP1	**0.42** (0.23–0.80)			
Total SBP1	**0.38** (0.20–0.72)			

Odds ratios (OR) and 95% confidence intervals (CI) for prostate cancer recurrence by quartile of SBP1 expression. Expression levels were measured using the VECTRA quantitative imaging system, and all OR estimates are adjusted for PSA, Gleason grade, tumor stage, and patient age at diagnosis.

Since highly variable intracellular SBP1 location was frequently observed within individual cores, we assessed whether the ratio of nuclear to cytoplasmic SBP1 was related to the likelihood of biochemical recurrence. Patients were assigned to quartiles based on the SBP1 nuclear to cytoplasmic ratio, and estimated odds ratios and 95% confidence intervals for the association between prostate cancer recurrence and each quartile were obtained. There was no evidence for a significant association between the nuclear to cytoplasmic ratio of SBP1 and prostate cancer recurrence (Table 2). A follow up analysis of the percentage of SBP1 positive cells in a given core revealed a reduced likelyhood of recurrence among patients in the highest quartiles when compared with the lowest quartile. Total cell percent positivity was not significantly associated with recurrence (S1 Table). The positivity threshold was defined by visibly detectable intensity.

**Table 2:** There was no association between the nuclear to cytoplasmic ratio of SBP1 and prostate cancer recurrence.

	SBP1 levels by quartile			
	1 (Low)	2	3	4 (high)
	OR (95% CI)	OR (95% CI)	OR (95% CI)	OR (95% CI)
Nuc:Cyt SBP1	**1.00**	**1.61** (0.80, 3.26)	**0.93** (.44, 1.95)	**0.60** (0.27, 1.37)

Odds ratios (OR) and 95% confidence intervals (CI) for prostate cancer recurrence by quartile of nuclear to cytoplasmic SBP1 expression. Expression level was measured by the VECTRA quantitative imaging system, and all OR estimates are adjusted for PSA, Gleason grade, tumor stage, and patient age at diagnosis.

### Ectopic expression of SBP1 does not impact proliferation of HCT116 cells but inhibits anchorage independent growth

Prostate cancer recurrence after radical prostatectomy occurs because primary tumors have already metastasized prior to surgery. Anchorage independent growth has been validated as a surrogate phenotype of metastatic potential using gene expression signatures [18]. In order to investigate SBP1’s impact on anchorage independent growth, a doxycycline-inducible SBP1 expression construct was introduced into HCT116 cells. These cells were chosen because of their lack of detectable SBP1 expression, the presence of a wild type p53 (see below) and because they have been used previously in studying SBP1 function [19]. S1A Fig shows the robust induction of SBP1 in HCT116 TetSBP1 cells.

**Fig 4:**
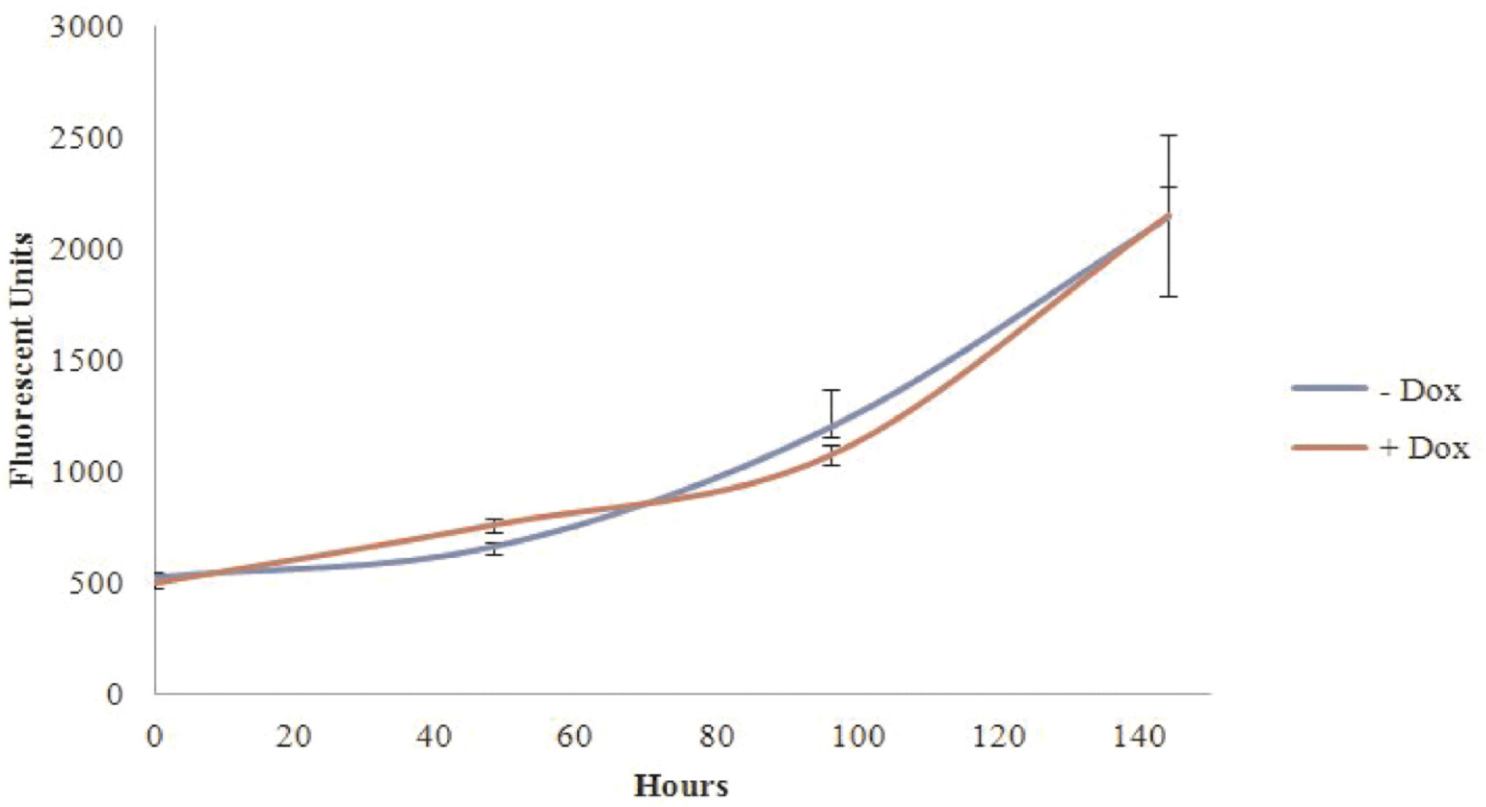
SBP1 does not change the proliferation of HCT116 cells. HCT116-TetSBP1 cells with (+Dox) and without (-Dox) SBP1 were grown for 6 days on a 96 well plate. Cells were treated with 1ug/mL doxycycline 48 hours before the 0 hour time point. Five hundred cells were seeded in triplicate 24 hours before the 0 hour time point. Fluorescently labeled dsDNA from each well of a 96 well plate was quantified at four time points- 0, 48, 96, and 144 hours, and error bars represent standard deviations at each time point.

**Fig 5:**
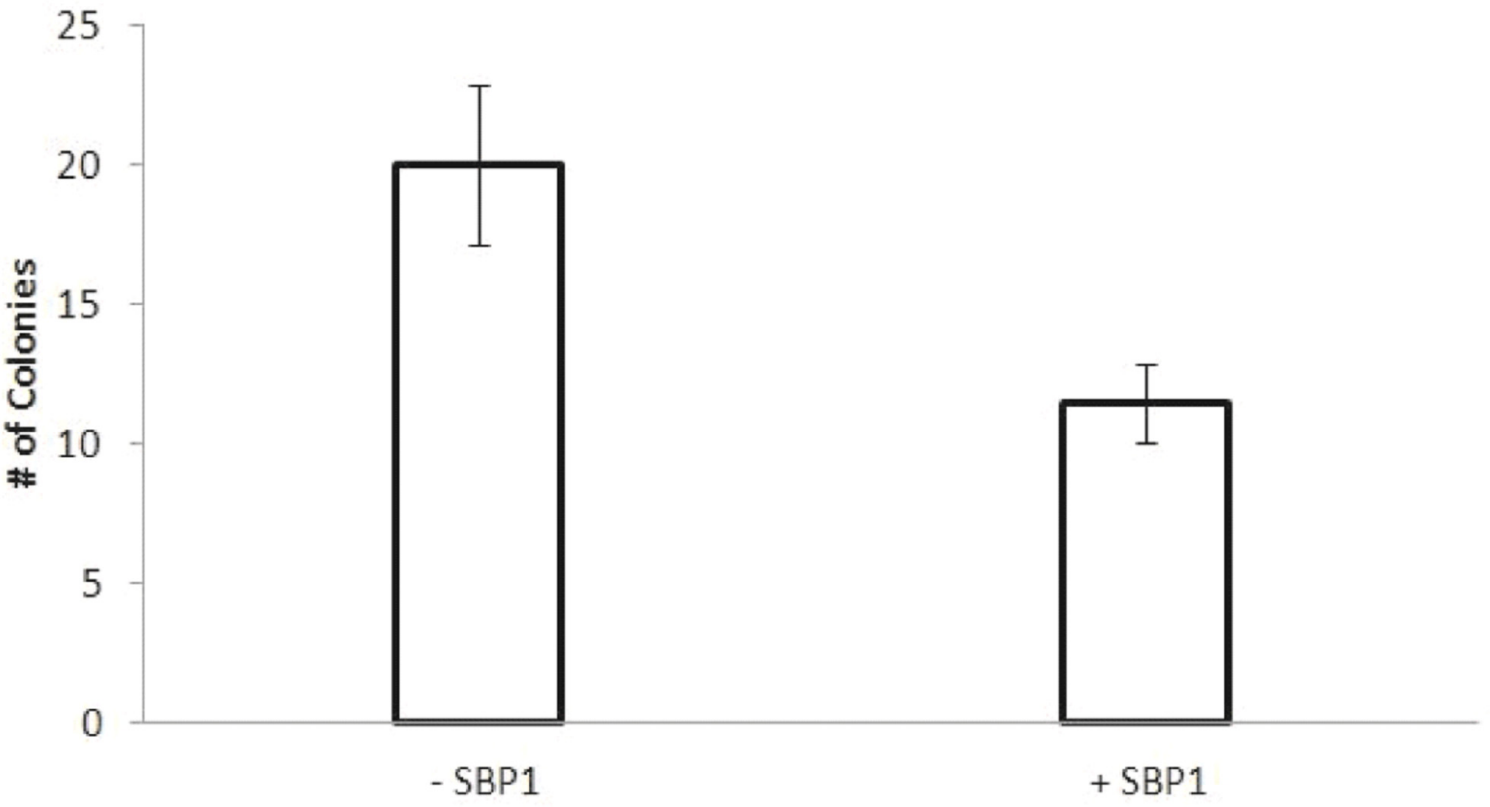
SBP1 decreases soft agar colony formation in HCT116 cells. The ability of HCT116 cells with and without SBP1 to form colonies in soft agar was measured. Cells induced to express SBP1 as well as SBP1-null cells were mixed with media containing 0.4% agarose, and each group was plated in triplicate on 6 well plates coated with 0.6% agarose in media. Each well contained 5x10^4^ cells, and colonies larger than 0.5mm were counted on day 15 of growth (A). Error bars represent S.D. (*p<0.05 n = 3).

The proliferation of HCT116 cells was not significantly affected by SBP1 expression (**Fig 4**). HCT116 cells which only contain the pRetroX-Tight-Pur-SBP1 plasmid without the TetOn transactivator plasmid were used to control for doxycycline effects on proliferation and were grown concurrently with the TetSBP1 cells, using the difference in growth between control cells with and without doxycycline at each time to adjust the TetSBP1 proliferation data. In order to do this, the fluorescent reported growth of doxycycline treated HCT116-TetSBP1 cells (fluorescent units, FU) was divided by the difference in FU seen between doxycycline treated and non-treated control cells. To determine the ability of SBP1 to impact growth in semi-solid media, HCT116-TetSBP1 cells expressing SBP1 (+Dox) as well as SBP1-null (-Dox) cells were suspended in 0.4% agarose, incubated, and colonies enumerated 15 days after plating. SBP1 significantly reduced the ability of the cells to grow in semi-solid media (**Fig 5**)

### SBP1 does not change expression of Glutathione Peroxidases-1 & 4, Thioredoxin Reductase-1 or NF-ĸB

One potential explanation for the suppressive effect of SBP1 on anchorage independent growth is that it affects the levels of selenoproteins which have been implicated in oncogenic signaling via altering the levels of reactive oxygen species (ROS). To investigate this possibility, lysates from HCT116 TetSBP1 cells with and without SBP1 induction were immunoblotted using antibodies against TrxRD1, GPx-1, and GPx-4. These three selenoproteins are antioxidants implicated in the etiology of several cancers [20]. SBP1 did not change the levels of any of the selenoproteins examined (S1B and S1C Fig), nor did it affect the levels of the ROS sensitive transcription factor NF-ĸB (S1A Fig).

### SBP1 sensitizes cells to 5-Fluorouracil

**Fig 6:**
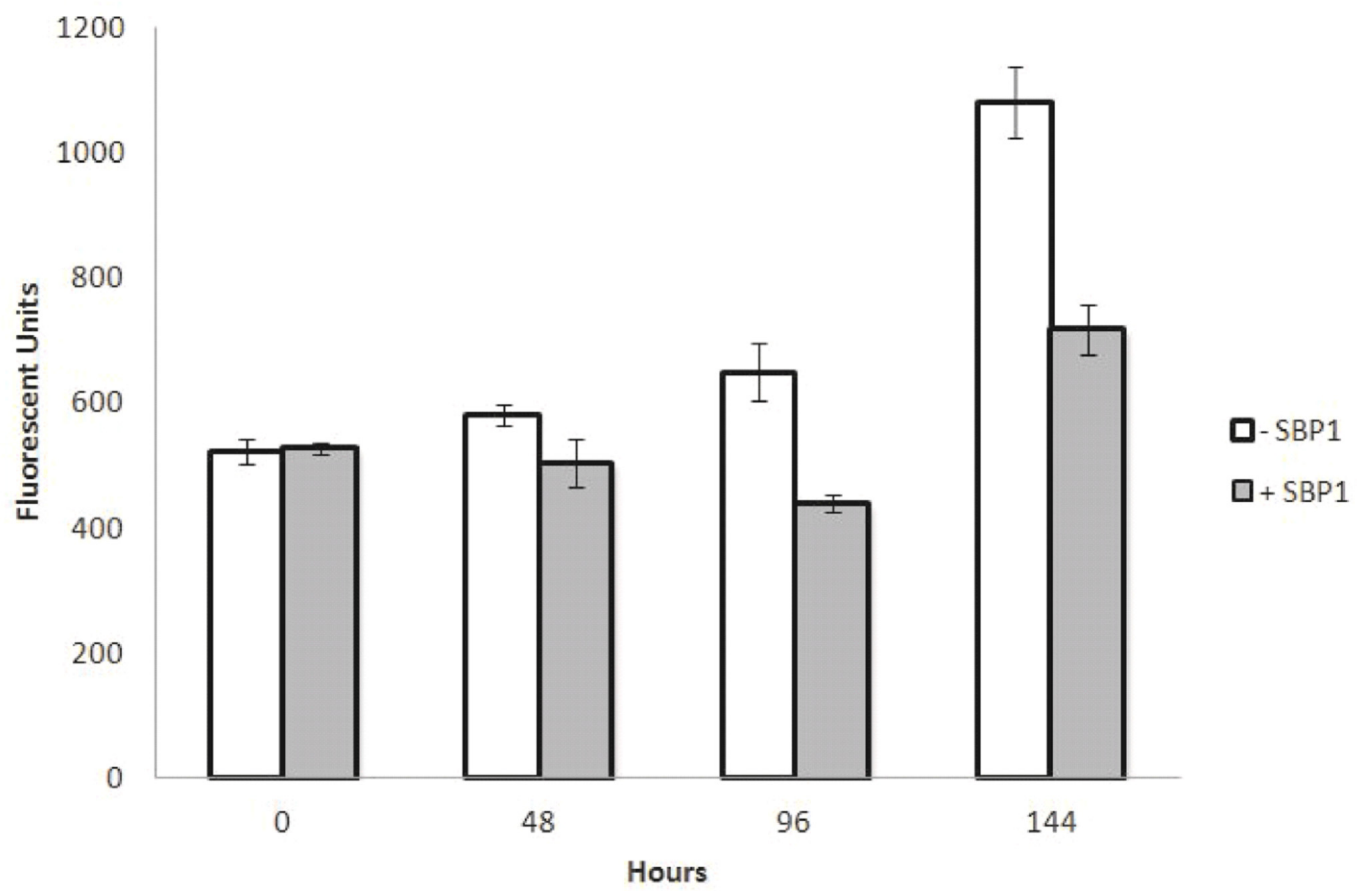
SBP1 sensitizes cells to treatment with colon cancer chemotherapeutic 5-FU. The growth of HCT116-TetSBP1 cells was measured after a 6 day treatment with 5uM 5-FU. Double stranded DNA was measured as an indicator of cell growth at four time points- 0, 48, 96, and 144 hours, and error bars represent standard deviation at each time point (*p<0.05, **p < 0.01). Five hundred cells were seeded in triplicate 24 hours before 0 hour time point, and fluorescently labeled dsDNA from each well of a 96 well plate was quantified after cells were lysed while still attached to the plate.

Another possible explanation for the association between aggressive prostate cancer and low nuclear SBP1 is that tumor cells, which typically experience high chromosomal instability, have survival advantage when there is low nuclear SBP1. In order to investigate if SBP1 affected cell response to DNA damage, HCT116 cells were treated with 5-Fluorouracil (5-FU), which blocks the synthesis of thymidine and leads to DNA damage [21,22]. SBP1 was induced in HCT116 TetSBP1 cells with doxycycline and grown on 96-well plates for 6 days and sampled at 48 hour time points concurrently with SBP1-null control cells at the same 5uM concentration of 5-FU. Cells expressing SBP1 were more sensitive to 5-FU exposure (Fig 6).

### SBP1 induces phosphorylation of p53 at serine 15

**Fig 7:**
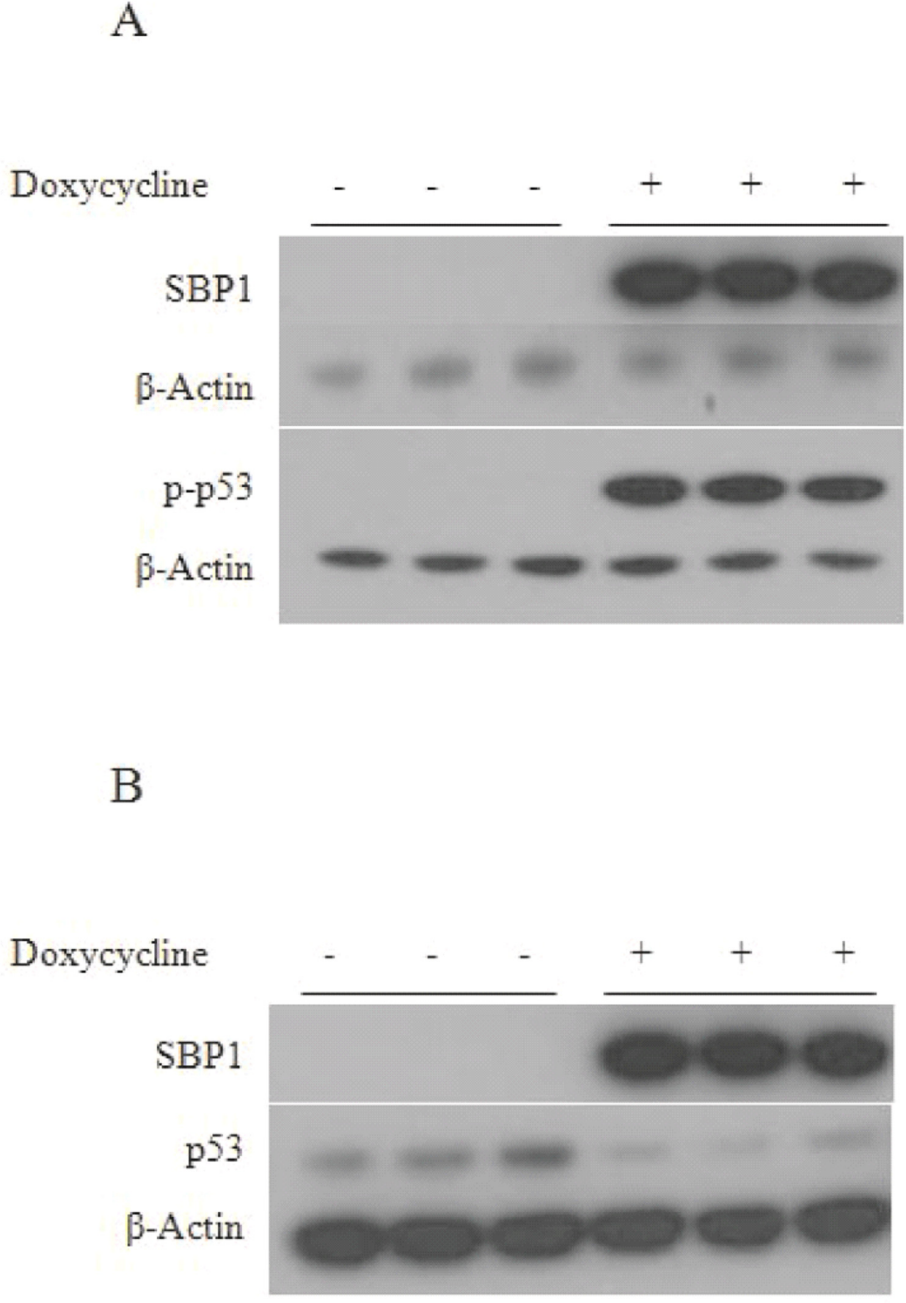
SBP1 induction results in an increase in phospho-p53 and a decrease in total p53 in HCT116 cells. Total cell extracts from doxycycline treated or non-treated HCT116-TetSBP1 cells were analyzed using immunobloting for changes in phosphorylated Ser15 on p53 (A) and total p53 (B) levels in response to induction of SBP1 using anti-human SBP1, phospho p53-Ser15, p53, and β-Actin antibodies. β-Actin was used as an endogenous control.

Previous work has described the involvement of p53 in the cellular response to 5-FU [23,24]. Given the ability of SBP1 to sensitize cells to 5-FU, we investigated whether SBP1 affects the phosphorylation of serine 15 (Ser15) on p53, which is a post-translational modification required to activate the p53-dependent DNA damage response [25]. Western blotting of extracts prepared from HCT116 cells did not detect phosphorylated p53-Ser15 (Fig 7A). However, induction of SBP1 resulted in robust phosphorylation of p53 on Ser15 (Fig 7A). It is apparent that the stimulation of the phosphorylation of p53 at Ser15 coincided with a decrease of total p53 (Fig 7B).

## Discussion

The association between SBP1 and prostate cancer aggressiveness was investigated because, previous studies reported lower SBP1 levels were associated with poor clinical outcome of patients with several other cancer types (reviewed in [26]). The association between SBP1 levels and several clinical features of prostate cancer was examined using prostate cancer tissue microarrays designed to identify variables that impact patient outcome. When a possible association between SBP1 levels and Gleason score was investigated, total SBP1 levels were not associated with Gleason score, consistent with our previous western blot examination of 24 prostatectomy specimen samples [27]. However, in the data present in this manuscript, there was a statistically significant lower SBP1 nuclear:cytoplasmic ratio in tissues categorized as higher grade prostate cancer (Category 3) compared to tissues categorized as lower grade (Category 1). In the subsequent analysis of biochemical recurrence, the cellular location of SBP1 was also used in the analysis of the tissue array, indicating that patients in the lowest quartile of nuclear SBP1 expression were significantly more likely to recur than those in any other quartile. The mechanistic significance of the cellular compartmentalization of SBP1 remains unknown, and no known enzymatic activity has been attributed to this protein. It is possible however, that changes in SBP1’s reported interaction and possible modulating affects on non-nuclear proteins such as GPx-1 and VDU1, contribute to SBP1’s biological function(s).

In order to investigate the molecular mechanisms by which SBP1 might impact cancer aggressiveness, we ectopically expressed it in cultured cells which do not express any detectable SBP1. Induction of SBP1 in these cells reduced their ability to grow in semi-solid media while not impacting the cells’ proliferative properties. While SBP1 has previously been shown to suppress anchorage independent growth in both PC3 and LNCaP human prostate tumor-derived cell lines, SBP1 inhibited the proliferation only of PC3 and not LNCaP cells [6]. As part of our efforts to gain an understanding of the mechanism by which SBP1 caused the observed phenotypes, a robust induction in the phosphorylation of p53 on Ser15 was observed (Fig **5**). As recently expanded upon, phosphorylation of p53 on Ser15 can impact a wide host of p53-mediated functions, including cell cycle arrest, as well as the response to DNA damage and other stresses [28]. It is therefore conceivable that at least some of the consequences of the loss of SBP1 expression that often accompanies malignant progression could be mediated through a reduction of p53 phosphorylation at this site, and the different effects of SBP1 on HCT116, PC3 and LNCaP may be due to the different status of p53 in those cells; HCT116 and LNCaP have functional p53 while PC3 do not [29,30]. Similarly, p53 dependent processes may be involved in the increase in sensitivity to 5-FU observed when HCT116 cells were induced to express SBP1. Using multicellular spheroids to study 5-FU resistance in colorectal cancer cells, Lee et al. reported that SBP1 was differentially expressed in 5-FU resistant cells and these authors suggested that SBP1 may be a novel biomarker of 5-FU chemoresistance [31]. The data presented herein, along with previous studies that have reported on the effects of SBP1 in cultured cells or the frequent loss of SBP1 in human cancers, collectively support the notion of a contributing role of SBP1 loss in cancer progression.

Dietary intake of selenium has been associated with reduced risk of prostate cancer in human epidemiological studies [32], although the results of the SELECT selenium supplementation trial established that providing low doses of selenium to older men at average risk in North America did not reduce prostate cancer incidence [33]. The mechanism by which dietary intake of selenium could conceivably prevent prostate cancer remains unknown, but it may involve selenium’s affects on selenium-containing proteins, several of which have been implicated in the risk of cancer and other diseases due to the association of specific genetic polymorphisms with disease risk [34,35]. The effects of selenium consumption on SBP1 or any of the selenocysteine-containing selenoproteins remain unknown. However, information regarding the likelihood of prostate cancer patients recurring after prostatectomy is of critical importance in the endeavor to prevent unnecessary treatment for men with indolent tumors. Future studies should consider the prognostic utility of examining SBP1 levels in prostate tissue and further investigate whether SBP1 loss is contributing to disease progression.

## Supporting Information

S1 Fig SBP1 expression does not change the levels of GPx-1, GPx-4, NF-ĸB or TrxRD1. Total cell extracts from doxycycline treated or non-treated HCT116-TetSBP1 cells were analyzed using immunoblot for changes in NF-ĸB (A) and TrxRD1 (B) levels in response to doxycycline dependent induction of SBP1. Anti-human SBP1, TrxRD1, NF-ĸB and β-Actin antibodies were used to detect protein levels. β-Actin was used as an endogenous control. Control cells only contain the pRetroX-SBP1 plasmid without transactivator.
(TIF)

S1 Table Prostate cancer patients with higher percentage of positive nuclear and cytoplasmic SBP1 staining in their prostate tumors were less likely to recur. Odds ratios (OR) and 95% confidence intervals (CI) for prostate cancer recurrence by quartile of percentage of positive nuclear and cytoplasmic SBP1 staining. Positivity was defined by visibly detectable intensity, and quantified using the measurements obtained by the VECTRA quantitative imaging system. All OR estimates are adjusted for PSA, Gleason grade, tumor stage, and patient age at diagnosis.
(DOCX)
