# Supplementary figures and images for: Evidence That Selenium Binding Protein 1 Is a Tumor Suppressor in Prostate Cancer
Source: PLoS One. 2015 May 18;10(5):e0127295. doi: 10.1371/journal.pone.0127295 (PMC4436248; doi:10.1371/journal.pone.0127295)

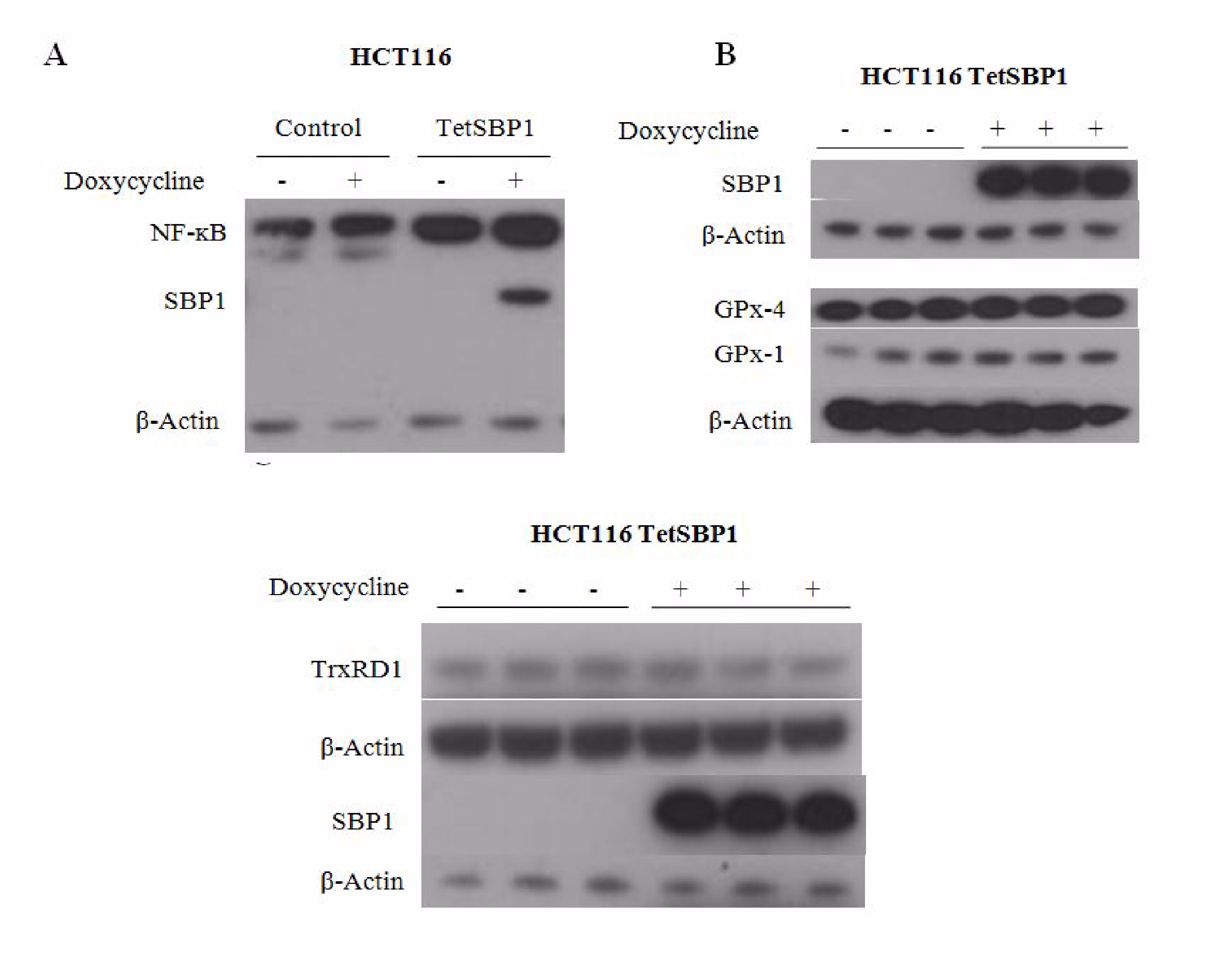

Supplement: S1 Fig — Total cell extracts from doxycycline treated or non-treated HCT116-TetSBP1 cells were analyzed using immunoblot for changes in NF-ĸB (A) and TrxRD1 (B) levels in response to doxycycline dependent induction of SBP1. Anti-human SBP1, TrxRD1, NF-ĸB and β-Actin antibodies were used to detect protein levels. β-Actin was used as an endogenous control. Control cells only contain the pRetroX-SBP1 plasmid without transactivator. (TIF) [file pone.0127295.s001.tif]
