# Supplementary material for: Evidence That Selenium Binding Protein 1 Is a Tumor Suppressor in Prostate Cancer
Source: PLoS One. 2015 May 18;10(5):e0127295. doi: 10.1371/journal.pone.0127295 (PMC4436248; doi:10.1371/journal.pone.0127295)
Supplement: S1 Table — Odds ratios (OR) and 95% confidence intervals (CI) for prostate cancer recurrence by quartile of percentage of positive nuclear and cytoplasmic SBP1 staining. Positivity was defined by visibly detectable intensity, and quantified using the measurements obtained by the VECTRA quantitative imaging system. All OR estimates are adjusted for PSA, Gleason grade, tumor stage, and patient age at diagnosis. (DOCX) [file pone.0127295.s002.docx]

|  | **Quartile 2-4 vs. 1** |
| --- | --- |
|  | **OR (**95% CI**)** |
| Nuc % Pos SBP1 | **0.51** (0.27 - 0.99) |
| Cyt % Pos SBP1 | **0.50** (0.26 - 0.94) |
| Total % Pos SBP1 | **0.64** (0.34 - 1.19) |
